# Immune Response to COVID-19 Vaccines: Updates in a Fast-Moving Scenario

**DOI:** 10.3390/vaccines14020168

**Published:** 2026-02-12

**Authors:** Federico De Marco

**Affiliations:** Gene Expression and Cancer Model Unit, IRCCS Regina Elena National Cancer Institute, 00144 Rome, Italy; federico.demarco@ifo.it or dott.federico.demarco@pec.it; Tel.: +39-339-1319160; Fax: +39-06-5266-2794

**Keywords:** COVID-19 vaccines, BNT162b2, moderna 1273, AZD1222, BBIBP-CorV, CoronaVac, Ad26.COV2-S, autoimmune rheumatic diseases, transplantation recipient, patients living with HIV

## Abstract

The prompt and extensive use of COVID-19 vaccines has dramatically reduced both cases and casualties, but it has also underscored a number of critical issues that need to be addressed for their full exploitation at global level. This Special Issue was launched to gather fresh data to improve the way we use vaccines that are currently available to contribute to the design and the development of new ones and to elucidate social, ethical and psychological concerns that might hamper vaccine acceptance and use. It includes 15 articles six of which address, under various aspects, the level and durability of protective immunity; five deal with the highly debated field of vaccine use, efficacy and safety, in persons with an impaired/dysregulated immune system; one paper reports on adjuvants’ role in immune stimulation; one on the interference of natural adenovirus immunity with DNA vaccine response; one is a review on the cellular mechanisms of vaccine-dependent myocarditis; and one explores social attitudes to vaccines in different ethnic groups during early phases of the pandemic. Far from being complete and exhaustive, this Special Issue provides the reader with fresh insights into several critical questions raised by or connected to the advent of COVID-19 vaccines. This introductory article provides an overview of their contribution, while offering a quick glance at the current state of the art.

## 1. Introduction

The SARS-CoV-2/COVID-19 outbreak in late 2019 elicited an unprecedented, huge international scientific effort, which resulted in the release of many differently formulated vaccines, much sooner than ever expected. Despite their extensive use, which has dramatically reduced the pandemic burden and attenuated clinical severity, a number of critical issues concerning their optimal use in the real world soon emerged. Namely:

(1) A dramatic gap became evident between the large body of experimental data available and the lack of sound, evidence-based indications to guide the field use of vaccines in the real world.
(2) Concerns about their safety and efficacy were raised due to their accelerated authorization while severe, although rare, adverse effects were alarmingly reported.
(3) The protective immunity elicited by COVID-19 vaccines turned out to be disappointingly short-lived.
(4) The taking over of viral variants and their noticeably different pathological profiles caused doubt towards the actual utility of the currently available vaccines.
(5) The black wave of No-Vax, as well as hesitancy/skepticism gained new momentum at a global level.

The fifteen manuscripts included here [1,2,3,4,5,6,7,8,9,10,11,12,13,14,15] aim to deepen our knowledge on aspects of the above issues.

## 2. Level and Durability of Protective Immunity

### 2.1. Determinants of Immune Response

The paper by Bates JA et al. [1] reports on the correlation between the Ab response and the body mass index (BMI) following complete basal immunization with the BNT162b2 mRNA-based vaccine (the Pfizer vaccine). A total of 126 adult healthy health care workers (HCW) with BMIs of 19 < 25; 25 < 30; 30 < 35; and >35 was assayed at roughly 50 and 200 days post the second dose, and the serum’s total receptor-binding-domain (RBD)-specific Ab and total serum neutralizing activity were measured. The results of this article show that BMI, which is statistically linked to severe clinical course, has no significant impact on BNT162b2-elicited Ab titers, suggesting the observed higher morbidity and mortality reported among people with a higher BMI is to be related to mechanisms other than an impaired immune reaction. As an adjunct, this paper also reports that the BNT162b2 vaccine induces a much higher Ab response compared with natural infection, which is a shared, potentially interesting observation that has not received the deserved attention so far.

The work of I Voulgaridi et al. [7] compares the anti-spike IgG and IgA responses induced by the prime vaccination with either BNT162b2, AZD1222 or Ad26.COV2. In this comparatively large study (*n* = 270), anti-spike IgG and IgA responses were evaluated on days 21, 42, 90, and 180 following the first dose in age- and sex-matched recipients. Their results indicate that both mRNA and adenovirus vector-based vaccines caused mild side-effects and were effective in inducing adequate antibody responses against SARS-CoV-2, although BNT162b2 was superior, concerning the intensity of antibody responses and protection against severe COVID-19. The intensity of the IgG and IgA responses depended primarily on both history of previous COVID-19 infection and the vaccination platform used, with individuals immunized with a single-dose vaccine having lower antibody titers over time.

The paper by Mudenda et al. [12] provides an interesting indication for the amelioration of the outcome of currently available DNA vaccines, showing that oral selenium supplementation in the experimental animal [(BALB/c mice (*n* = 18)] can generate more robust immune responses with almost no or minimal general and metabolic toxicity, thus paving the way to further studies on accessories’ components in vaccine responses.

Along the same lines is paper [15] by J Kim et al., reporting data on the outcome of the adenovirus (Ad)-derived ChAdOx1 nCoV-19 vaccine, according to pre-existing Ad immunity. Ad-derived vectors are very promising tools in gene therapy, vaccine development and in cancer research. Indeed, they can efficiently transduce many proliferating and quiescent cell types, can accommodate large amounts of exogenous DNA, rarely integrate into the host genome and are amenable to large-scale production. Regrettably, adenovirus infections are highly incident among the general population and preexisting immunity may lead to early vector clearance, poor tissue transduction and possible immune-dependent adverse reactions (see references in [15]). With their paper, Kim J et al. contribute precious real-world data showing that pre-existing adenovirus immunity could be identified in 49 of 68 adult patients (72.1%) vaccinated with two doses of the ChAdOx1 nCoV-19. They also show that the geometric mean titer of S-specific IgG antibodies was statistically higher in individuals without pre-existing Ad immunity, among whom reactogenicity also had a higher frequency in comparison with patients with pre-existing Ad immunity.

### 2.2. Durability of Vaccinal Protection

The paper by Roeder AJ et al. [8] compares the durability of Neutralizing Ab titers, measured in a surrogate LFA test, after the second and third dose of either of the mRNA-based vaccines, BNT162b2 and Moderna1273, in a total of 265 patients post-second dose and in roughly half of them (142) post-third dose too. Accurate statistical analysis data also provides clues about age-based, sex-based differences and mixed boosting schedules indicating that, irrespective of the manufacturer, a third dose of an mRNA vaccine provides NAb titers that are higher and more durable than a second dose (up to 8 months versus hardly 4 months). The data also show that Ab elicited by a third dose, encoding the original sequence of the Wuhan-Hu-1 SARS-CoV-2 isolate, protects against severe disease and hospitalization with Omicron variants, but not against infection and mild symptomatic disease. In addition, a mixed primary/boosting schedule seems to offer marginal improvements compared with homologous schedules, and the Moderna-1273 induce a slightly longer persistence. Based on their results, the authors propose to distinct patients into three types: namely, vaccine strong responder, vaccine mild responder and vaccine poor responder.

The impact of a third booster dose of the BNT162b2 vaccine is further addressed by the contribution of Assavavongwaikit P et al. [5]. In this cross-sectional study on 116 adolescents (a class that is known to have a higher immune response to the BNT162b2 vaccine than young adults), they report that the third dose considerably raised the titers of total anti RBD Ab as well as of NAb against both the Delta and Omicron variant, as measured by a surrogate ACE2/RBD binding inhibition assay and confirmed by a pseudo-type viral neutralization assay. In addition, they also showed that, in a selected group of sera, before the third dose administration (i.e., over 5 months post-second dose administration), the anti-Delta surrogate Nab titers were still in the protection range, while those against the Omicron variant were close to the baseline, thus confirming Roeder et al.’s findings that at least a 3-dose regimen is necessary to elicit fair protection against the Omicron variant, even in the highly respondent class of adolescents. This contribution also deals with vaccine acceptance/hesitancy, showing that Thai adolescents in the high-income population range are largely favorable to receive booster doses, as they are correctly aware of vaccine efficacy, eager to protect their families and partners from the risk of spreading infection, and keen to safely resume social activities.

### 2.3. Heterologous/Homologous Vaccine Regimen

During the early phases of the pandemic, because of the urgency of the situation and the shortage of the vaccine supply, the opportunity/necessity of adopting alternative off-label schedules and regimens (e.g., heterologous schemes) was intensely debated. In their study, Kim DI and co-workers [11] provide a comparative analysis of mRNA- and vector-based vaccine platforms assessing spike-binding IgG levels and neutralizing capacity in 66 vaccinated individuals immunized either by homologous (BNT162b2/BNT162b2 or ChAdOx1/ChAdOx1) or heterologous (ChAdOx1-BNT162b2) regimen. Their results indicate that a stronger induction and a comparatively rapid waning of antibodies was consequent to the homologous RNA vaccination, while a weaker response and a more persistent humoral response (i.e., over 6 months) was observed in the homologous DNA vaccine group. Finally, the heterologous vaccination with ChAdOx1 and BNT162b2 resulted in an effective boost effect, with the highest remaining antibody responses being at six months post-primary vaccination.

Again, the contribution by Chansaenroj J et al. [4] reports the Ab response kinetics in healthy individuals who were primed with the inactivated BBIBP-CorV vaccine to a third heterologous boosting dose, either consisting of BNT162b2, 1273 or AZD122. Serum total anti-RBD Ig and anti-RBD IgG were measured by electro-chemio-luminescence immunoassay (ECLIA) and by chemiluminescent microparticle immunoassay (CMIA), respectively. Neutralizing Anti-RBD Ab was measured by a surrogate RBD/ACE2 binding inhibition assay. Specific RBD CD4+ and CD8+ T cell responses were evaluated by the QuantiFERON ELISA Assay. In agreement and extending the above-mentioned results [5], these authors confirm the disappointingly short duration of anti RBD serum positivity induced by the basic two-shot vaccination. In addition, they provide evidence that, in BBIBP primed persons, the heterologous booster with either BNT162b2 or 1273 elicited higher total anti-RBD lg and anti-RBD lgG than the one induced by AZD1222. In agreement with other previous studies, they also show that the booster with any of the three formulations protects adequately against the prototype virus, as well as the Delta variant, while just a short-lived protection was conferred against the 0-Micron variant, with the durability granted by BNT162b2 being much longer than those by 1273 and AZD122, respectively.

## 3. Vaccine Use in Frail Persons

Five communications focus on the highly controversial field of vaccine use, efficacy and safety in frail persons. Indeed, people with co-morbidities are the most at risk for infectious complications and, at the same time, they present a higher level of potential counter-indications and a poorer unsuccessful response. The papers included here deal with: patients living with HIV (PLWH) [3,6], patients undergoing systemic anti-neoplastic treatments [2], recipients of allogeneic hematopoietic stem cell transplantation (HSCT) [10] and patients with autoimmune rheumatic diseases [14].

The work by G Zeng et al. [3] reports about the immunogenicity and the humoral immune persistence following prime vaccination with two doses of either the BBIBP-CorV (Sinopharm) or CoronaVac (SinoVac) vaccine in 132 adult PLWH. Their results show that in PLWH, the S-RBD-IgG Ab seropositivity rates and levels were lower than in healthy controls (HCs) and their decline was faster, thus indicating that two doses of inactivated vaccine may not be enough to provide PLWH with reasonable persisting protective immunity, suggesting the routine use of at least three dose regimens.

The impact of a third boosting dose in PLWH was then investigated, in a BNT162b2 homologous setting, by L Gianserra et al. [6]. These authors monitored 42 subjects for anti-spike antibodies, CD4+ and CD8 counts and viraemia at time zero, T0 (pre-vaccination), T1 (4 weeks after the second dose), T2 (pre-booster) and T3 (4 weeks after the booster dose). The results were then correlated to sex, age, BMI, and nadir and baseline CD4+ counts, as well as the type of cART regimen. Their findings indicate that Ab levels elicited by the booster dose were sharply higher compared with those of all the other time points and that sex, age, BMI, CD4+ counts, and cART regimen did not significantly affect the antibody response induced by the booster dose. Thus, the BNT162b2 boosting vaccination in PLWH is safe and greatly enhances the immune response mounted by the primary vaccination.

Another condition, that is possibly responsible for an inadequate immune response to vaccines, is systemic antineoplastic therapy. Indeed, irrespective of cancer’s histological type, disease stage and grade and therapeutic protocols, antineoplastic treatments have the potential for hampering/suppressing the coordinated cellular and humoral responses needed by protective immune reactions. To shed light on such a critical question, AT Waickman and co-workers [2] assessed vaccine-elicited immunity in a cohort of patients with advanced solid tumors either under observation or receiving systemic anti-cancer therapy. Their analysis reveals that both cellular and humoral immunity in individuals with cancer who were receiving systemic anti-cancer therapy were not significantly different from those of individuals under observation. Furthermore, even though some patients exhibited suboptimal antibody titers, specific cellular immune responses were still detected, showing that serum Ab titers offer an incomplete picture of vaccine-elicited immunity in cancer patients and that specific cellular immunity does exist, even in the absence of a significant humoral response.

M Watanabe et al. [10] evaluated the efficacy and safety of a third dose of homologous BNT162b2 vaccination in a small group (*n* = 22) of allogeneic hematopoietic stem cell transplantation (HSCT) patients. Despite the very small number of patients taken into account, a number of stimulating observations were made: first, all but one of these HSCT patients (95%) achieved seroconversion after the third dose, including patients taking immunosuppressants or steroids; second, although some patients had lost seroconversion before the third dose, the antibody titers after the third dose were much higher than after the second dose for all patients except one; third, five of the six HSCT patients who had not obtained immunogenicity after the second dose obtained seroconversion after the third inoculation. Finally, none of these patients had serious adverse events, such as new-onset graft-versus-host disease (GVHD) or GVHD exacerbation. As a matter of fact, this latter observation is partially conflicting with those of other groups, which is possibly because of the small sample or ethnicity factor in the assayed sample; nonetheless, they demonstrate that at least the BNT162b2 vaccine is safe and effective for allogeneic HSCT patients, to whom a boosting dose is clearly indicated.

Intapiboon P et al [14] provide information about the outcome of mRNA heterologous booster in patients suffering with autoimmune rheumatic diseases (ARDs) and vaccine primed with either a complete heterologous (CoronaVac/ChAdOx1) or a homologous (ChAdOx1/ChAdOx1) schedule. Measuring the anti-SARS-CoV-2 receptor binding domain (RBD) IgG levels at one and three months after mRNA booster vaccination these authors showed the satisfactory humoral immunogenicity of mRNA vaccine boosters after a primary series with vaccine strategies other than the mRNA platform while just minor disease flare-ups occurred in 18.2% of the patients.

## 4. Vaccine Toxicity and Adverse Effects

The accelerated release of many different COVID-19 vaccines brought about a dramatic improvement in the pandemic crisis. However, while quite successful in controlling the spreading and casualties of COVID-19, several concerns about their long-term health risks and adverse effects have been raised, due to their unprecedentedly short development cycle. The paper by KR Hamedi et al. [13] provides us with a comprehensive review of the highly complex mechanisms of viral myocarditis: a rare, yet potentially fatal, condition caused by both viral infections and vaccinations. This review compares the partially distinct pathways of viral myocarditis with those believed to be involved in vaccine-associated myocarditis and discuss this topic in relation to the congenital heart defect patient population, a subset of patients at increased risk of the invalidating/life-threatening long-term complications of myocarditis. The authors show how prolonged and uncontrolled cytokine-mediated inflammation and autoantibody formation may increase the risk for the cardiac fibrotic remodeling that may persist despite the resolution of myocarditis. For a complex and delicate balance, the authors conclude stating that, while some caution is warranted due to risk of cardiac damage, the risk of serious morbidity and mortality from COVID-19, including COVID-19 viral myocarditis, largely outweigh the risks of COVID-19 vaccine-associated myocarditis in the CHD population.

## 5. Vaccine Social Acceptance and Hesitancy

Together with scientifically based concerns, a plethora of prejudicial, malicious or openly fraudulent critics were raised about vaccines and COVID-19 vaccines, and a tidal wave of skepticisms and hesitancy severely impacted the public opinions worldwide. The manuscript by Z Wang and co-workers [9], utilizing geo-tweets and Bayesian-based methods, investigates the spatiotemporal changes in public engagement and public sentiment to COVID-19 vaccines and how public engagement and sentiment react to different vaccine-related topics in various racial groups in the USA. They show that, in general, the public took a positive attitude towards vaccination and that public sentiment positivity increased as an increasingly larger number of people were vaccinated. However, public sentiment on specific topics varied in different periods, and skepticism was slightly more prevalent among African Americans compared with other ethnic groups.

## 6. Conclusions

This Special Issue, although far from being complete and definitive, provides the reader with further insight into several critical questions raised by or connected to the advent of COVID-19 vaccines. I do hope it will be of help to the many researchers and clinicians engaged in the vaccine-related control of infectious diseases. Finally, I would like to thank all the authors, reviewers, assistants, and editors who made this work possible, both at viruses and in the wider academic community.

## Abbreviations

The following abbreviations are used in this manuscript:

1273	The mRNA-based Spikevax vaccine (by Moderna)
Ab	Antibodies
Ad	Adenovirus
Ad26.COV2-S	The single shot DNA vaccine by Janssen Biontech
ARDs	Autoimmune rheumatic Diseases
AZD122	The ChAdOx1 DNA-vectored AstraZeneca vaccine
BBIBP-CorV	The Sinopharm subunit inactivated vaccine
BMI	Body mass index
BNT162b2	Comirnatry (the Pfitzer mRNA Vaccine)
cART	Combination antiretroviral therapy
CoronaVac	The Sinovac subunit inactivated vaccine
GVHD	Graft-versus-host disease (GVHD),
HCs	Healthy controls
HCW	Health care workers
HSCT	Allogenic hematopoietic stem cell transplantation
NAb	Neutralizing antibodies
PLWH	Patients living with HIV
RBD	Receptor-binding-domain
S-RBD-IgG	Anti Spike receptor binding domain-specific IgG Antibodies
